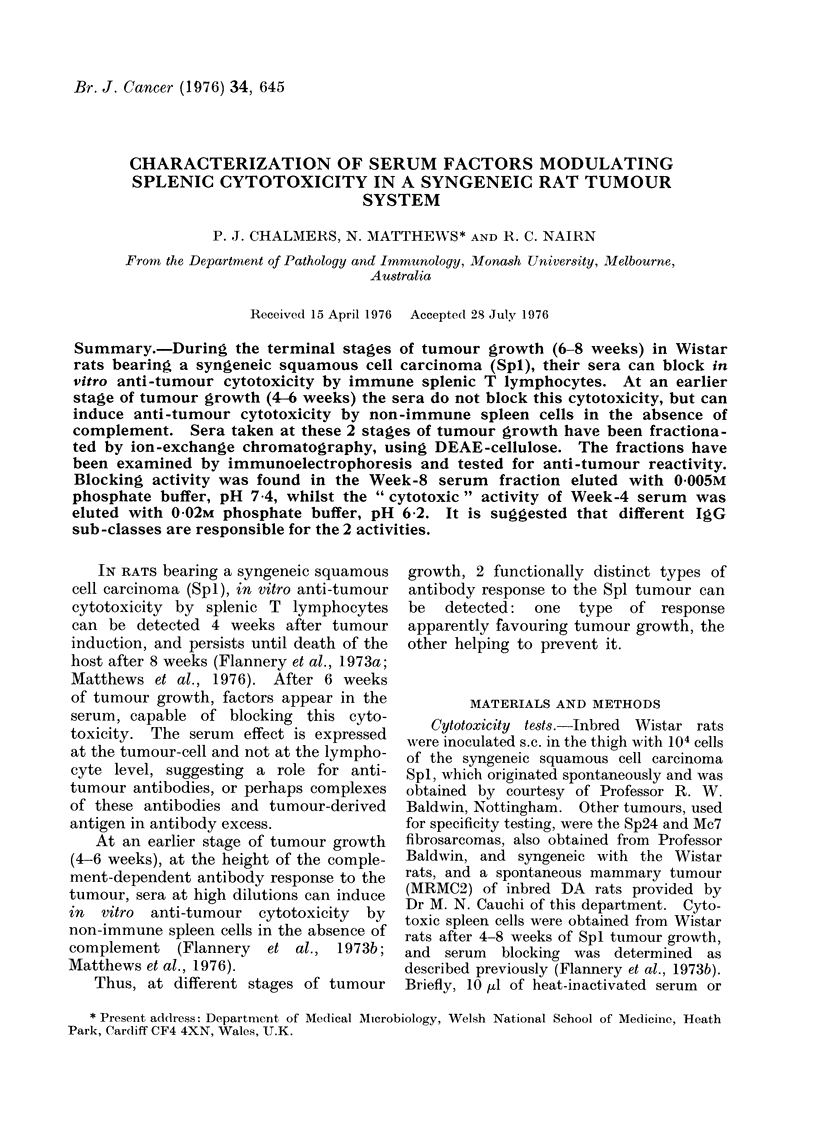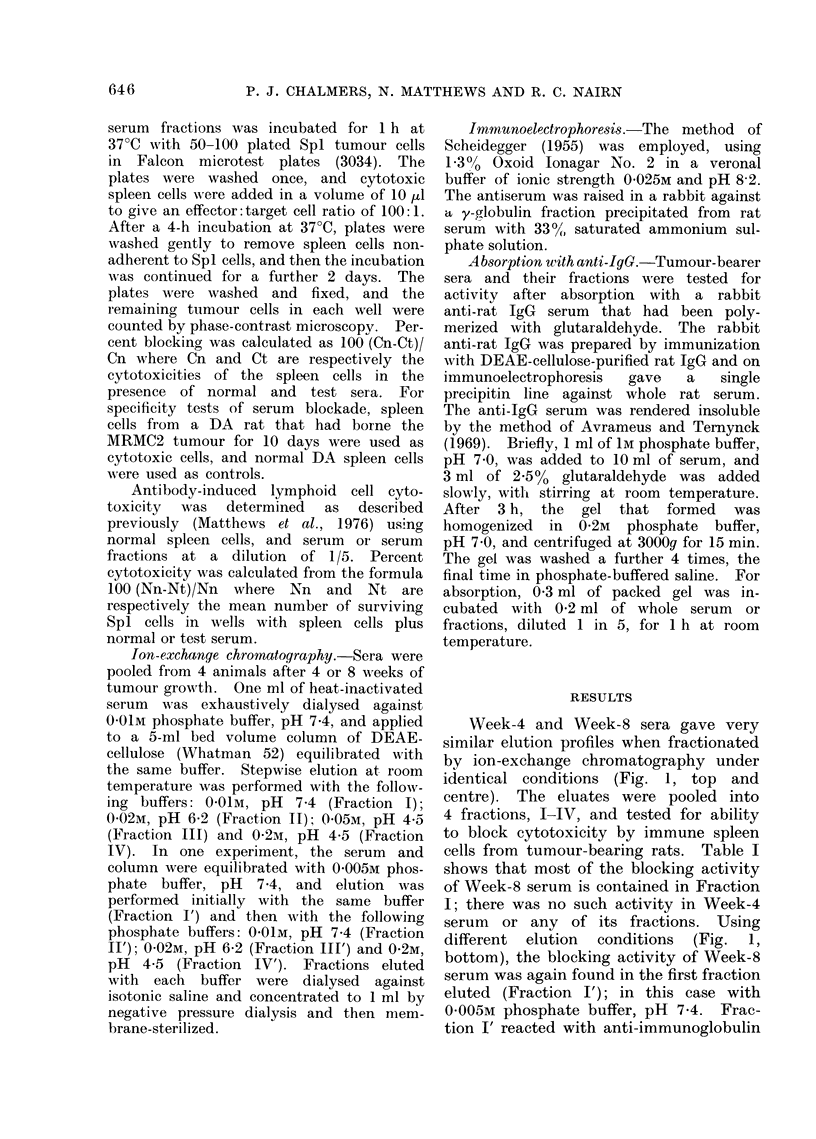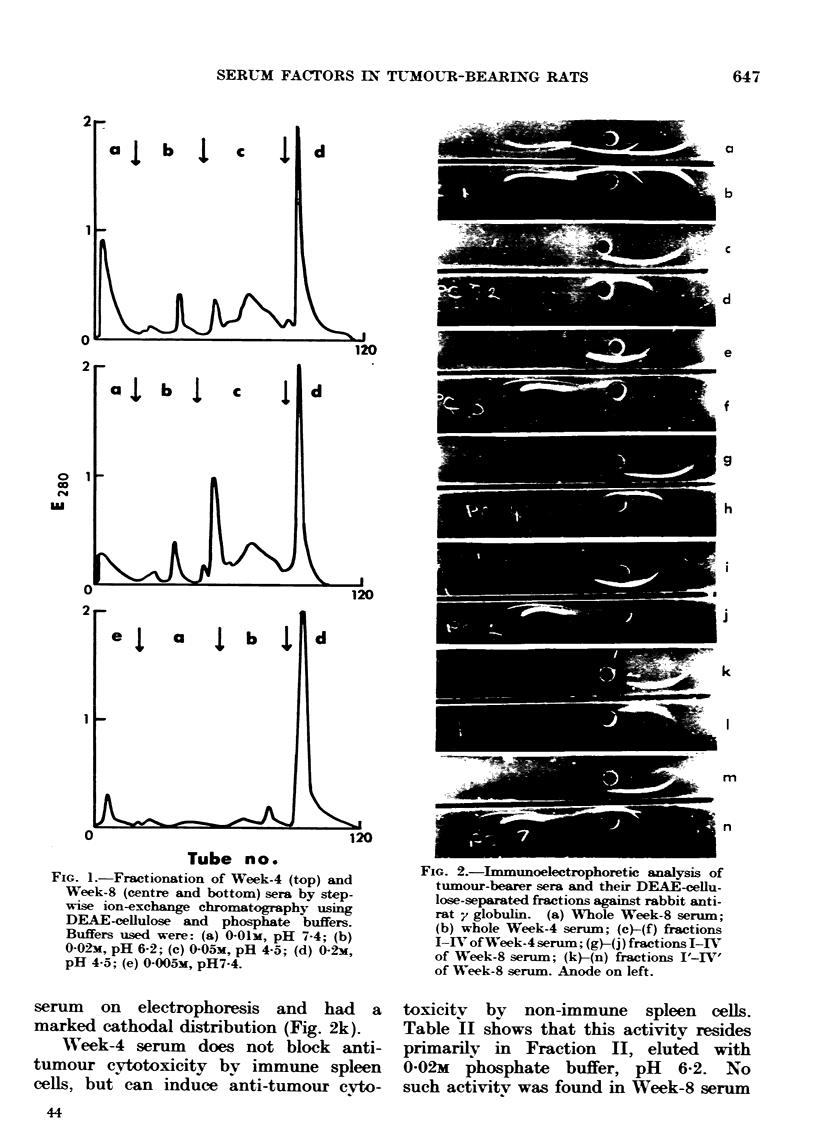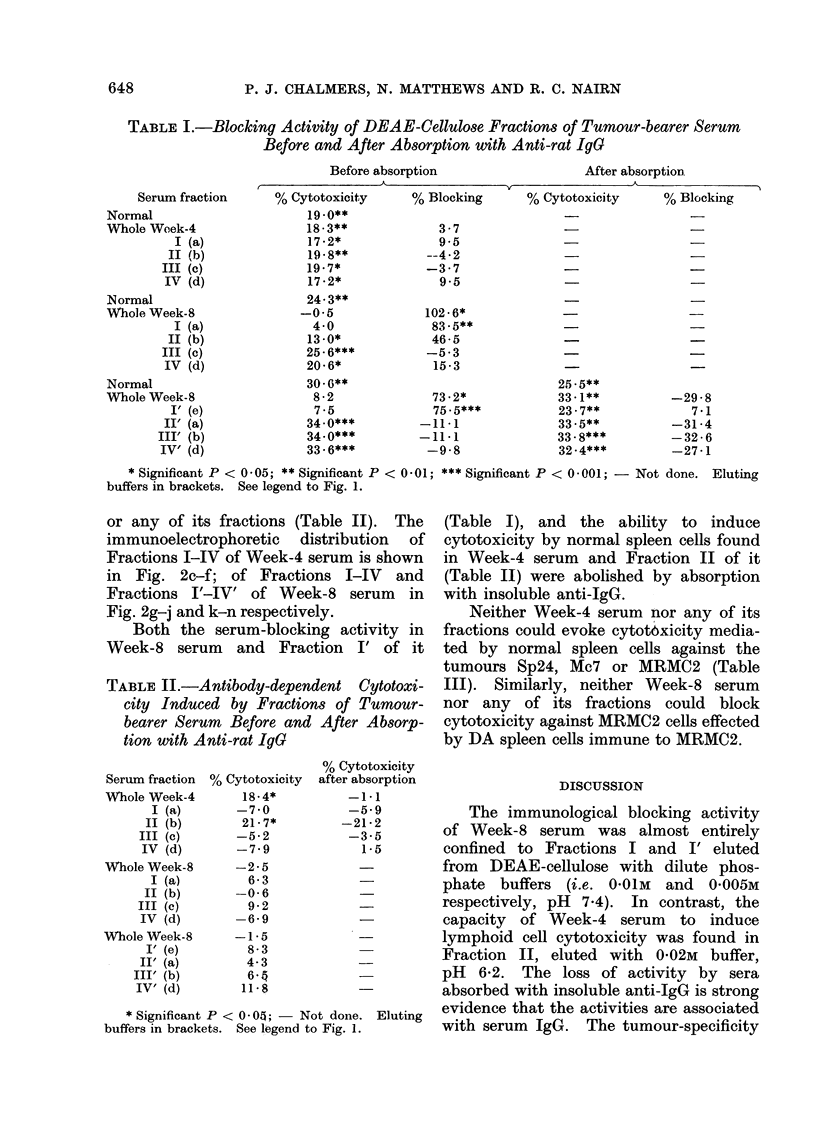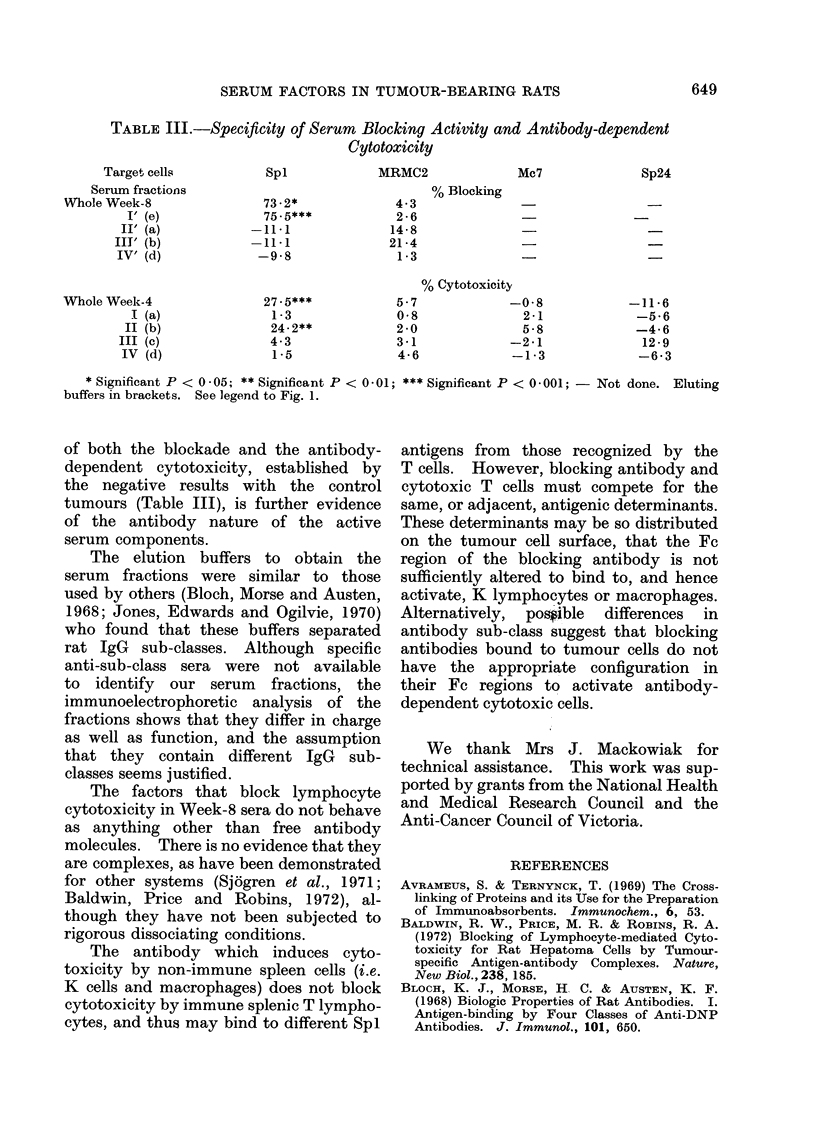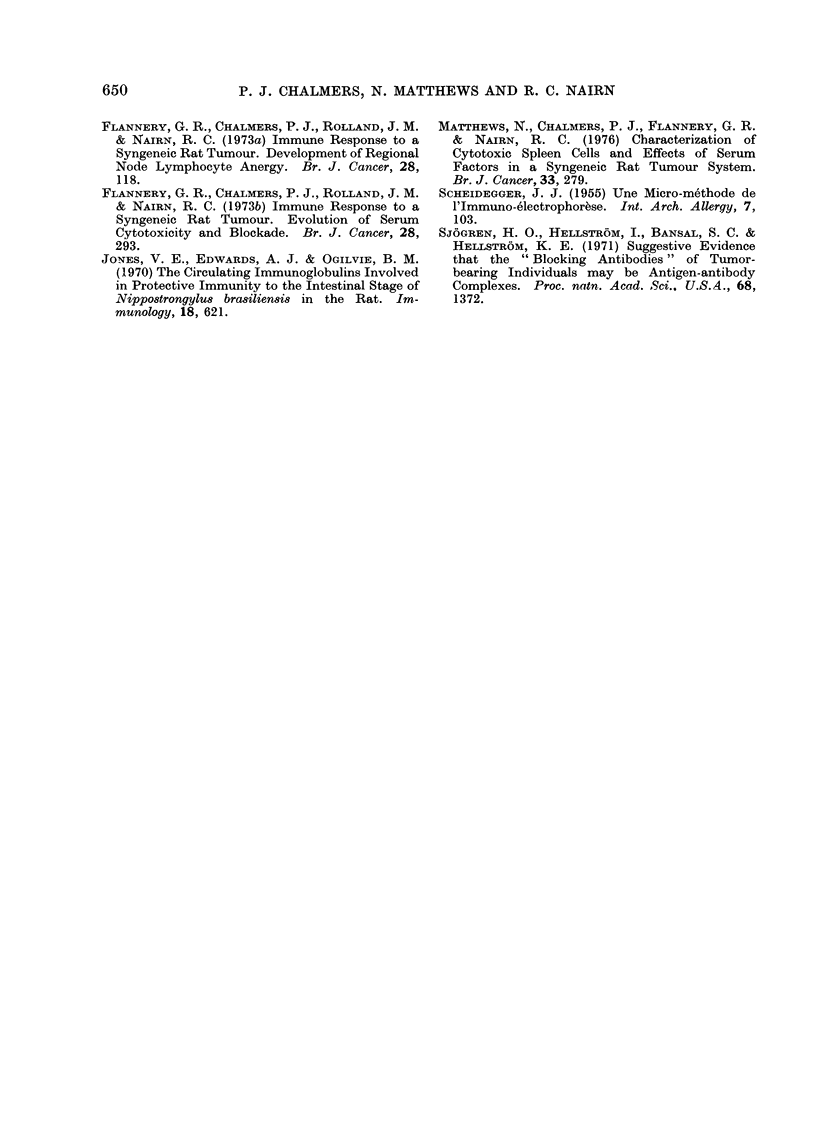# Characterization of serum factors modulating splenic cytotoxicity in a syngeneic rat tumour system.

**DOI:** 10.1038/bjc.1976.226

**Published:** 1976-12

**Authors:** P. J. Chalmers, N. Matthews, R. C. Nairn

## Abstract

**Images:**


*Br. J. Cancer* (1976) **34**, 645

### CHARACTERIZATION OF SERUM FACTORS MODULATING SPLENIC CYTOTOXICITY IN A SYNGENEIC RAT TUMOUR SYSTEM

P. J. CHALMERS, N. MATTHEWS* AND R. C. NAIRN

*From the Department of Pathology and Immunology, Monash University, Melbourne, Australia*

Received 15 April 1976 Accepted 28 July 1976

**Summary.—During the terminal stages of tumour growth (6–8 weeks) in Wistar rats bearing a syngeneic squamous cell carcinoma (Spl), their sera can block *in vitro* anti-tumour cytotoxicity by immune splenic T lymphocytes. At an earlier stage of tumour growth (4–6 weeks) the sera do not block this cytotoxicity, but can induce anti-tumour cytotoxicity by non-immune spleen cells in the absence of complement. Sera taken at these 2 stages of tumour growth have been fractionated by ion-exchange chromatography, using DEAE-cellulose. The fractions have been examined by immunoelectrophoresis and tested for anti-tumour reactivity. Blocking activity was found in the Week-8 serum fraction eluted with 0·005M phosphate buffer, pH 7·4, whilst the "cytotoxic" activity of Week-4 serum was eluted with 0·02M phosphate buffer, pH 6·2. It is suggested that different IgG sub-classes are responsible for the 2 activities.**

IN RATS bearing a syngeneic squamous cell carcinoma (Spl), *in vitro* anti-tumour cytotoxicity by splenic T lymphocytes can be detected 4 weeks after tumour induction, and persists until death of the host after 8 weeks (Flannery *et al.*, 1973*a*; Matthews *et al.*, 1976). After 6 weeks of tumour growth, factors appear in the serum, capable of blocking this cytotoxicity. The serum effect is expressed at the tumour-cell and not at the lymphocyte level, suggesting a role for anti-tumour antibodies, or perhaps complexes of these antibodies and tumour-derived antigen in antibody excess.

At an earlier stage of tumour growth (4–6 weeks), at the height of the complement-dependent antibody response to the tumour, sera at high dilutions can induce *in vitro* anti-tumour cytotoxicity by non-immune spleen cells in the absence of complement (Flannery *et al.*, 1973*b*; Matthews *et al.*, 1976).

Thus, at different stages of tumour growth, 2 functionally distinct types of antibody response to the Spl tumour can be detected: one type of response apparently favouring tumour growth, the other helping to prevent it.

### MATERIALS AND METHODS

*Cytotoxicity tests.*—Inbred Wistar rats were inoculated s.c. in the thigh with $10^4$ cells of the syngeneic squamous cell carcinoma Spl, which originated spontaneously and was obtained by courtesy of Professor R. W. Baldwin, Nottingham. Other tumours, used for specificity testing, were the Sp24 and Mc7 fibrosarcomas, also obtained from Professor Baldwin, and syngeneic with the Wistar rats, and a spontaneous mammary tumour (MRMC2) of inbred DA rats provided by Dr M. N. Cauchi of this department. Cytotoxic spleen cells were obtained from Wistar rats after 4–8 weeks of Spl tumour growth, and serum blocking was determined as described previously (Flannery *et al.*, 1973*b*). Briefly, 10 $\mu$l of heat-inactivated serum or

* Present address: Department of Medical Microbiology, Welsh National School of Medicine, Heath Park, Cardiff CF4 4XN, Wales, U.K.

serum fractions was incubated for 1 h at 37°C with 50–100 plated Spl tumour cells in Falcon microtest plates (3034). The plates were washed once, and cytotoxic spleen cells were added in a volume of 10 $\mu$l to give an effector: target cell ratio of 100:1. After a 4-h incubation at 37°C, plates were washed gently to remove spleen cells non-adherent to Spl cells, and then the incubation was continued for a further 2 days. The plates were washed and fixed, and the remaining tumour cells in each well were counted by phase-contrast microscopy. Percent blocking was calculated as 100 (Cn-Ct)/Cn where Cn and Ct are respectively the cytotoxicities of the spleen cells in the presence of normal and test sera. For specificity tests of serum blockade, spleen cells from a DA rat that had borne the MRMC2 tumour for 10 days were used as cytotoxic cells, and normal DA spleen cells were used as controls.

Antibody-induced lymphoid cell cytotoxicity was determined as described previously (Matthews *et al.*, 1976) using normal spleen cells, and serum or serum fractions at a dilution of 1/5. Percent cytotoxicity was calculated from the formula 100 (Nn-Nt)/Nn where Nn and Nt are respectively the mean number of surviving Spl cells in wells with spleen cells plus normal or test serum.

*Ion-exchange chromatography.*—Sera were pooled from 4 animals after 4 or 8 weeks of tumour growth. One ml of heat-inactivated serum was exhaustively dialysed against 0·01M phosphate buffer, pH 7·4, and applied to a 5-ml bed volume column of DEAE-cellulose (Whatman 52) equilibrated with the same buffer. Stepwise elution at room temperature was performed with the following buffers: 0·01M, pH 7·4 (Fraction I); 0·02M, pH 6·2 (Fraction II); 0·05M, pH 4·5 (Fraction III) and 0·2M, pH 4·5 (Fraction IV). In one experiment, the serum and column were equilibrated with 0·005M phosphate buffer, pH 7·4, and elution was performed initially with the same buffer (Fraction I′) and then with the following phosphate buffers: 0·01M, pH 7·4 (Fraction II′); 0·02M, pH 6·2 (Fraction III′) and 0·2M, pH 4·5 (Fraction IV′). Fractions eluted with each buffer were dialysed against isotonic saline and concentrated to 1 ml by negative pressure dialysis and then membrane-sterilized.

*Immunoelectrophoresis.*—The method of Scheidegger (1955) was employed, using 1·3% Oxoid Ionagar No. 2 in a veronal buffer of ionic strength 0·025M and pH 8·2. The antiserum was raised in a rabbit against a $\gamma$-globulin fraction precipitated from rat serum with 33% saturated ammonium sulphate solution.

*Absorption with anti-IgG.*—Tumour-bearer sera and their fractions were tested for activity after absorption with a rabbit anti-rat IgG serum that had been polymerized with glutaraldehyde. The rabbit anti-rat IgG was prepared by immunization with DEAE-cellulose-purified rat IgG and on immunoelectrophoresis gave a single precipitin line against whole rat serum. The anti-IgG serum was rendered insoluble by the method of Avrameus and Ternynck (1969). Briefly, 1 ml of 1M phosphate buffer, pH 7·0, was added to 10 ml of serum, and 3 ml of 2·5% glutaraldehyde was added slowly, with stirring at room temperature. After 3 h, the gel that formed was homogenized in 0·2M phosphate buffer, pH 7·0, and centrifuged at 3000*g* for 15 min. The gel was washed a further 4 times, the final time in phosphate-buffered saline. For absorption, 0·3 ml of packed gel was incubated with 0·2 ml of whole serum or fractions, diluted 1 in 5, for 1 h at room temperature.

### RESULTS

Week-4 and Week-8 sera gave very similar elution profiles when fractionated by ion-exchange chromatography under identical conditions (Fig. 1, top and centre). The eluates were pooled into 4 fractions, I–IV, and tested for ability to block cytotoxicity by immune spleen cells from tumour-bearing rats. Table I shows that most of the blocking activity of Week-8 serum is contained in Fraction I; there was no such activity in Week-4 serum or any of its fractions. Using different elution conditions (Fig. 1, bottom), the blocking activity of Week-8 serum was again found in the first fraction eluted (Fraction I′); in this case with 0·005M phosphate buffer, pH 7·4. Fraction I′ reacted with anti-immunoglobulin

FIG. 1.—Fractionation of Week-4 (top) and Week-8 (centre and bottom) sera by stepwise ion-exchange chromatography using DEAE-cellulose and phosphate buffers. Buffers used were: (a) 0·01M, pH 7·4; (b) 0·02M, pH 6·2; (c) 0·05M, pH 4·5; (d) 0·2M, pH 4·5; (e) 0·005M, pH7·4.

FIG. 2.—Immunoelectrophoretic analysis of tumour-bearer sera and their DEAE-cellulose-separated fractions against rabbit anti-rat $\gamma$ globulin. (a) Whole Week-8 serum; (b) whole Week-4 serum; (c)–(f) fractions I–IV of Week-4 serum; (g)–(j) fractions I–IV of Week-8 serum; (k)–(n) fractions I′–IV′ of Week-8 serum. Anode on left.

serum on electrophoresis and had a marked cathodal distribution (Fig. 2k).

Week-4 serum does not block anti-tumour cytotoxicity by immune spleen cells, but can induce anti-tumour cytotoxicity by non-immune spleen cells. Table II shows that this activity resides primarily in Fraction II, eluted with 0·02M phosphate buffer, pH 6·2. No such activity was found in Week-8 serum

TABLE I.—*Blocking Activity of DEAE-Cellulose Fractions of Tumour-bearer Serum Before and After Absorption with Anti-rat IgG*

| | Before absorption | | After absorption | |
|---|---|---|---|---|
| Serum fraction | % Cytotoxicity | % Blocking | % Cytotoxicity | % Blocking |
| Normal | 19·0** | | — | — |
| Whole Week-4 | 18·3** | 3·7 | — | — |
| I (a) | 17·2* | 9·5 | — | — |
| II (b) | 19·8** | --4·2 | — | — |
| III (c) | 19·7* | −3·7 | — | — |
| IV (d) | 17·2* | 9·5 | — | — |
| Normal | 24·3** | | — | — |
| Whole Week-8 | −0·5 | 102·6* | — | — |
| I (a) | 4·0 | 83·5** | — | — |
| II (b) | 13·0* | 46·5 | — | — |
| III (c) | 25·6*** | −5·3 | — | — |
| IV (d) | 20·6* | 15·3 | — | — |
| Normal | 30·6** | | 25·5** | |
| Whole Week-8 | 8·2 | 73·2* | 33·1** | −29·8 |
| I′ (e) | 7·5 | 75·5*** | 23·7** | 7·1 |
| II′ (a) | 34·0*** | −11·1 | 33·5** | −31·4 |
| III′ (b) | 34·0*** | −11·1 | 33·8*** | −32·6 |
| IV′ (d) | 33·6*** | −9·8 | 32·4*** | −27·1 |

* Significant $P < 0·05$; ** Significant $P < 0·01$; *** Significant $P < 0·001$; — Not done. Eluting buffers in brackets. See legend to Fig. 1.

or any of its fractions (Table II). The immunoelectrophoretic distribution of Fractions I–IV of Week-4 serum is shown in Fig. 2c–f; of Fractions I–IV and Fractions I′–IV′ of Week-8 serum in Fig. 2g–j and k–n respectively.

Both the serum-blocking activity in Week-8 serum and Fraction I′ of it (Table I), and the ability to induce cytotoxicity by normal spleen cells found in Week-4 serum and Fraction II of it (Table II) were abolished by absorption with insoluble anti-IgG.

TABLE II.—*Antibody-dependent Cytotoxicity Induced by Fractions of Tumour-bearer Serum Before and After Absorption with Anti-rat IgG*

| Serum fraction | % Cytotoxicity | % Cytotoxicity after absorption |
|---|---|---|
| Whole Week-4 | 18·4* | −1·1 |
| I (a) | −7·0 | −5·9 |
| II (b) | 21·7* | −21·2 |
| III (c) | −5·2 | −3·5 |
| IV (d) | −7·9 | 1·5 |
| Whole Week-8 | −2·5 | — |
| I (a) | 6·3 | — |
| II (b) | −0·6 | — |
| III (c) | 9·2 | — |
| IV (d) | −6·9 | — |
| Whole Week-8 | −1·5 | — |
| I′ (e) | 8·3 | — |
| II′ (a) | 4·3 | — |
| III′ (b) | 6·5 | — |
| IV′ (d) | 11·8 | — |

* Significant $P < 0·05$; — Not done. Eluting buffers in brackets. See legend to Fig. 1.

Neither Week-4 serum nor any of its fractions could evoke cytotoxicity mediated by normal spleen cells against the tumours Sp24, Mc7 or MRMC2 (Table III). Similarly, neither Week-8 serum nor any of its fractions could block cytotoxicity against MRMC2 cells effected by DA spleen cells immune to MRMC2.

### DISCUSSION

The immunological blocking activity of Week-8 serum was almost entirely confined to Fractions I and I′ eluted from DEAE-cellulose with dilute phosphate buffers (*i.e.* 0·01M and 0·005M respectively, pH 7·4). In contrast, the capacity of Week-4 serum to induce lymphoid cell cytotoxicity was found in Fraction II, eluted with 0·02M buffer, pH 6·2. The loss of activity by sera absorbed with insoluble anti-IgG is strong evidence that the activities are associated with serum IgG. The tumour-specificity

TABLE III.—*Specificity of Serum Blocking Activity and Antibody-dependent Cytotoxicity*

| Target cells | Spl | MRMC2 | Mc7 | Sp24 |
|---|---|---|---|---|
| Serum fractions | | % Blocking | | |
| Whole Week-8 | 73·2* | 4·3 | — | — |
| I′ (e) | 75·5*** | 2·6 | — | — |
| II′ (a) | −11·1 | 14·8 | — | — |
| III′ (b) | −11·1 | 21·4 | — | — |
| IV′ (d) | −9·8 | 1·3 | — | — |
| | | % Cytotoxicity | | |
| Whole Week-4 | 27·5*** | 5·7 | −0·8 | −11·6 |
| I (a) | 1·3 | 0·8 | 2·1 | −5·6 |
| II (b) | 24·2** | 2·0 | 5·8 | −4·6 |
| III (c) | 4·3 | 3·1 | −2·1 | 12·9 |
| IV (d) | 1·5 | 4·6 | −1·3 | −6·3 |

* Significant $P < 0{\cdot}05$; ** Significant $P < 0{\cdot}01$; *** Significant $P < 0{\cdot}001$; — Not done. Eluting buffers in brackets. See legend to Fig. 1.

of both the blockade and the antibody-dependent cytotoxicity, established by the negative results with the control tumours (Table III), is further evidence of the antibody nature of the active serum components.

The elution buffers to obtain the serum fractions were similar to those used by others (Bloch, Morse and Austen, 1968; Jones, Edwards and Ogilvie, 1970) who found that these buffers separated rat IgG sub-classes. Although specific anti-sub-class sera were not available to identify our serum fractions, the immunoelectrophoretic analysis of the fractions shows that they differ in charge as well as function, and the assumption that they contain different IgG sub-classes seems justified.

The factors that block lymphocyte cytotoxicity in Week-8 sera do not behave as anything other than free antibody molecules. There is no evidence that they are complexes, as have been demonstrated for other systems (Sjögren *et al.*, 1971; Baldwin, Price and Robins, 1972), although they have not been subjected to rigorous dissociating conditions.

The antibody which induces cytotoxicity by non-immune spleen cells (*i.e.* K cells and macrophages) does not block cytotoxicity by immune splenic T lymphocytes, and thus may bind to different Spl antigens from those recognized by the T cells. However, blocking antibody and cytotoxic T cells must compete for the same, or adjacent, antigenic determinants. These determinants may be so distributed on the tumour cell surface, that the Fc region of the blocking antibody is not sufficiently altered to bind to, and hence activate, K lymphocytes or macrophages. Alternatively, possible differences in antibody sub-class suggest that blocking antibodies bound to tumour cells do not have the appropriate configuration in their Fc regions to activate antibody-dependent cytotoxic cells.

We thank Mrs J. Mackowiak for technical assistance. This work was supported by grants from the National Health and Medical Research Council and the Anti-Cancer Council of Victoria.

### REFERENCES

AVRAMEUS, S. & TERNYNCK, T. (1969) The Cross-linking of Proteins and its Use for the Preparation of Immunoabsorbents. *Immunochem.*, **6**, 53.

BALDWIN, R. W., PRICE, M. R. & ROBINS, R. A. (1972) Blocking of Lymphocyte-mediated Cytotoxicity for Rat Hepatoma Cells by Tumour-specific Antigen-antibody Complexes. *Nature, New Biol.*, **238**, 185.

BLOCH, K. J., MORSE, H. C. & AUSTEN, K. F. (1968) Biologic Properties of Rat Antibodies. I. Antigen-binding by Four Classes of Anti-DNP Antibodies. *J. Immunol.*, **101**, 650.

FLANNERY, G. R., CHALMERS, P. J., ROLLAND, J. M. & NAIRN, R. C. (1973*a*) Immune Response to a Syngeneic Rat Tumour. Development of Regional Node Lymphocyte Anergy. *Br. J. Cancer*, **28**, 118.

FLANNERY, G. R., CHALMERS, P. J., ROLLAND, J. M. & NAIRN, R. C. (1973*b*) Immune Response to a Syngeneic Rat Tumour. Evolution of Serum Cytotoxicity and Blockade. *Br. J. Cancer*, **28**, 293.

JONES, V. E., EDWARDS, A. J. & OGILVIE, B. M. (1970) The Circulating Immunoglobulins Involved in Protective Immunity to the Intestinal Stage of *Nippostrongylus brasiliensis* in the Rat. *Immunology*, **18**, 621.

MATTHEWS, N., CHALMERS, P. J., FLANNERY, G. R. & NAIRN, R. C. (1976) Characterization of Cytotoxic Spleen Cells and Effects of Serum Factors in a Syngeneic Rat Tumour System. *Br. J. Cancer*, **33**, 279.

SCHEIDEGGER, J. J. (1955) Une Micro-méthode de l'Immuno-électrophorèse. *Int. Arch. Allergy*, **7**, 103.

SJÖGREN, H. O., HELLSTRÖM, I., BANSAL, S. C. & HELLSTRÖM, K. E. (1971) Suggestive Evidence that the "Blocking Antibodies" of Tumor-bearing Individuals may be Antigen-antibody Complexes. *Proc. natn. Acad. Sci., U.S.A.*, **68**, 1372.